# Vestibular assessment in children with sensorineural hearing loss: diagnostic accuracy and proposal for a diagnostic algorithm

**DOI:** 10.3389/fneur.2024.1349554

**Published:** 2024-02-01

**Authors:** Max Gerdsen, Tamara Maria Hundscheid, An Boudewyns, Vincent Van Rompaey, Raymond Van De Berg, Josine Christine Colette Widdershoven

**Affiliations:** ^1^Division of Vestibular Disorders, Department of Otorhinolaryngology and Head and Neck Surgery, Maastricht University Medical Center+, Maastricht, Netherlands; ^2^Department of Pediatrics, Maastricht University Medical Center+, Maastricht, Netherlands; ^3^Department of Otorhinolaryngology and Head and Neck Surgery, Antwerp University Hospital, Antwerp, Belgium; ^4^Faculty of Medicine and Translational Neurosciences, University of Antwerp, Antwerp, Belgium

**Keywords:** vestibular hypofunction, vestibular function, children, vestibular testing, video head impulse test, vestibular evoked myogenic potential, caloric test, rotatory chair test

## Abstract

**Introduction:**

Vestibular assessment in children with sensorineural hearing loss (SNHL) is critical for early vestibular rehabilitation therapy to promote (motor) development or guide decision making towards cochlear implantation (timing of surgery and laterality). It can be challenging from a clinical viewpoint to decide which vestibular tests should be performed for a pediatric patient. The aim of this study was to evaluate the diagnostic accuracy of several clinically available vestibular tests in children with SNHL, and to provide recommendations for the implementation of vestibular testing of children in clinical practice, to screen for vestibular hypofunction (VH).

**Methods:**

A two-center retrospective chart review was conducted. Eighty-six patients between the age of 0 and 18 years were included in this study with SNHL. Vestibular tests included video headimpulse test (VHIT), caloric test (performed at the age of four or higher), rotatory chair and cervical vestibular evoked myogenic potential (cVEMP). A combination of the clinical assessment and (combinations of) vestibular test outcomes determined the diagnosis. The diagnostic quality of tests and combination of tests was assessed by diagnostic accuracy, sensitivity and specificity.

**Results:**

VH was diagnosed in 44% of the patients. The VHIT and caloric test showed the highest diagnostic accuracy compared to the rotatory chair and cVEMP. All combinations of VHIT, caloric test and cVEMP showed improvement of the diagnostic accuracy compared to the respective tests when performed singularly. All combinations of tests showed a relatively similar diagnostic accuracy, with the VHIT combined with the caloric test scoring the highest. Adding a third test did not substantially improve the diagnostic accuracy.

**Discussion:**

Vestibular testing is feasible and VH is highly prevalent in children with SNHL. A proposed diagnostic algorithm recommends starting with VHIT, followed by cVEMP for children under the age of four, and caloric testing for older children if VH is not confirmed with the first test. Performing a third test is redundant as the diagnostic accuracy does not improve substantially. However, challenges remain, including the lack of a gold standard and the subjective nature of the diagnosis, highlighting the need for standardized testing and increased understanding of VH in this population.

## 1 Introduction

Children with sensorineural hearing loss (SNHL) are at risk of vestibular hypofunction (VH) due to the close anatomical and embryological relation between the cochlea and vestibular system. It seems evident that some causes of hearing loss also affect vestibular function. Reported prevalence of VH in children with SNHL ranges from 30 to 70% (1–6).

Pediatric VH is often unrecognized due to the limited communicative abilities of young children and atypical expression of vestibular symptoms such as frequent falls, delayed motor development milestones, impaired reading, writing or learning skills, or even delayed cognitive and socio-emotional development (2, 7–11). Vestibular assessment in children with SNHL is critical for early vestibular rehabilitation therapy to promote (motor) development. In addition, information on vestibular function may guide decision making towards cochlear implantation (timing of the surgery and laterality) or in the future for vestibular implantation (12–15).

Vestibular testing includes video head impulse testing (VHIT), caloric testing, rotary chair testing and cervical vestibular evoked myogenic potentials (cVEMP) (16, 17). It can be challenging from a clinical viewpoint to decide which vestibular tests should be performed and what modifications to improve reproducibility and tolerance should be made for a pediatric patient (18–21). Therefore, routinely testing vestibular function in children is unfortunately only performed in a limited number of centers around the world.

Previously, the results of these quantitative vestibular tests in children were compared. A high variability in prevalence of VH and diagnostic accuracy between studies and vestibular tests was found (22). Consequently, further research should be performed to reach consensus on a standard protocol for vestibular testing in children, preferably by performing all vestibular function tests in the same cohort, and using an adequate sample size. Therefore, the aim of this study was to evaluate the diagnostic accuracy of several clinically available vestibular tests in children with SNHL, and to provide recommendations for the implementation of vestibular testing of children in clinical practice, to screen for VH.

## 2 Methods

### 2.1 Patient selection

A retrospective chart review was conducted from January 2015 to May 2021 at the Maastricht University Medical Center and Antwerp University Hospital, after approval from the local Ethics Committees (2019–1,215, Edge 001056, respectively). Eighty-six patients between the age of 0 and 18 years were included in this study. Inclusion criteria were unilateral or bilateral sensorineural or mixed hearing loss, with a pure tone average of at least 30 decibels (dB) between 0.5 and 4 kilohertz in the unaided situation (hearing aid, cochlear implant).

### 2.2 Vestibular testing

During the outpatient visit, physical examination of the ear-, nose-, and throat area, and observations of motor function were performed in the examination room; these included stance, locomotion, one-leg-standing test (eyes open and eyes closed), Romberg test and an oculomotor evaluation. In addition, oculomotor testing was performed before vestibular testing. These tests comprised: smooth pursuit and range of eye movements, saccades, optokinetic nystagmus, spontaneous nystagmus and vergence. Subjects with signs of central vestibular pathology during oculomotor testing were excluded. Peripheral vestibular function was tested by VHIT, caloric test, rotatory chair and/or cVEMP. A minimum of two tests were executed in each patient to examine the vestibular function. The vestibular tests were performed in a randomized order to nullify potential confounding factors as fatigue or loss of attention by the patients per test. Vestibular tests were performed by different examiners, all trained in the execution of these tests in children. An otorhinolaryngologist (J.W.) with expertise in pediatric vestibular disorders and testing performed the clinical examination and was responsible for the interpretation of the vestibular tests in both centers.

VHIT testing was performed in both centers using the Synapsys system (Ulmer, Marseille, France) as previously described (23), or ICS impulse system (Otometrics-Natus, Taastrup, Denmark) for some patients above the age of 12. In case of the Synapsys system, a high-speed infrared camera was used to measure head and eye movements. The ICS impulse system used goggles, which included a high-speed infrared camera to measure eye movements, and gyroscopes to measure head movements. The head was moved in a horizontal plane with fast outward movements to the left and right (head impulses) while standing behind the patient to test the horizontal semicircular canal function (22, 24). To each side, at least 15 head impulses (under the age of four, minimum five head impulses) were applied with a peak head velocity between 150 and 400 °/s. A vestibular-ocular reflex gain below 0.8 was considered an abnormal response. Normative values were acquired at Maastricht University Medical Center from 39 healthy subjects aged 0–14 years (median 6.7 years; SD 4.2 years). The interpretation of the test results in both centers were compared to the normative data acquired in Maastricht University Medical Center.

Caloric testing was performed with the devices Variotherm 3 (Atmos, Lenzkirch, Germany; used in Maastricht University Medical Center) and KALORIstar Arctic 1 (Biomed, Jena, Germany; used in Antwerp University Hospital) as previously described (21). The patient laid in supine position with the head elevated at an angle of 30° in a dark room. Each ear was irrigated according to stimulus conditions with a water flow of 300 mL at 34 and 40°C for 30 s (Maastricht) or an air flow of 5–8 liters/min at 25 and 44°C (Antwerp) for 60 s to test the horizontal semicircular canal function. Response to irrigation was recorded for 5 min after irrigation. Due to the invasiveness of the caloric test, a minimum age of 4 years was applied. Caloric responses were compared to acquired data in healthy adults as the caloric response maturates before the aforementioned age that testing is performed (21). The asymmetry between vestibular systems in individuals was calculated with the Jongkees’ formula (25).

Rotary chair testing was performed using electrodes and the Nydiag 2000 motorized rotary chair (Ekida GmbH, Buggingen, Germany; used in Maastricht University Medical Center) or VESTAR 100 (Datmed, Wermelskirchen, Germany; used in Antwerp University Hospital) as previously described (6). The head was pitched forwardly at an angle of 30° and the chair turned in a sinusoidal motion for 60 s with a peak velocity of 60 degrees/s at a frequency of 0.1 Hz. Normative values were acquired from 63 healthy subjects aged 0–16 years at both centers (median 7.8 years; SD 5.3 years). The mean of the gain with standard deviation was applied to determine a normal or abnormal response per age category.

Eye movements during oculomotor testing, caloric testing and rotatory chair testing were measured by electronystagmography. Self-adhesive electrodes (White Sensor ECG electrodes, Ambu, Copenhagen, Denmark) were placed above the eyebrows, the inferior orbital margin of both eyes, the left and right exterior and interior canthi, and the reference electrode was placed on the forehead. Patients below the age of 2 years had a similar setup, except the electrodes were placed around one eye. Eye movements were recorded with electronystagmography (KingsLab 1.8.1, Maastricht University, Maastricht, The Netherlands, applied in Maastricht University Medical Center; Nystagliner, version 4.03, Toennies, Germany or BalanceLab, Maastricht Instruments BV, Maastricht, Netherlands, applied in Antwerp University Hospital).

Cervical VEMP testing was performed as previously described (6), using the Neuro-Audio DI200300 EMG System (Version 2010, Neurosoft, Ivanovo, Russia). Four self-adhesive electrodes (White Sensor ECG electrodes, Ambu, Copenhagen, Denmark) were placed on the belly of both sternocleidomastoid muscles, sternum (reference electrode) and forehead (ground electrode). A 59 decibels normalized hearing loss (129 dB sound pressure level) click was administered through a bone conductor on the mastoid at a frequency of 5 hertz for 100 clicks, to test the saccular function. The patient was placed in supine position, with the head elevated at an angle of 30^o^ from the horizontal plane and turned contralaterally to the stimulated ear to contract the sternocleidomastoid muscle. The patient was encouraged with the help of visual stimulation (e.g., viewing video on tablet, showing favorite toy). Each ear was tested at least twice for reproducibility. The interpretation of normal and abnormal cVEMP responses was conducted as described in Martens et al. (26). For short, responses were considered normal if at least two reproducible biphasic P1-N1 waveforms were observed. The amplitude of the waveforms needed to reach the normative cut-off value described in the aforementioned protocol to conclude a normal response. Additionally, the test result was considered abnormal when the latencies were prolonged, i.e., were greater than SD + 1 (P13 > 18.03 ms, N23 > 24.58 ms). The lower cut-off values (mean -1SD) were not considered. Normative data were acquired in healthy infants at a mean age of 7.6 months with the same protocol as performed in the aforementioned article.

### 2.3 Diagnostic accuracy

The outcome of each vestibular test was classified as “normal” or “abnormal” based on age-dependent normative ranges measured at the laboratories of Maastricht and Antwerp or literature as described previously. “Abnormal” indicated vestibular hypofunction. For VHIT, caloric test and VEMPs, results were interpreted for each vestibular organ separately. This implied that each patient was classified twice regarding vestibular hypofunction: each side could have vestibular hypofunction (or not). In case of rotatory chair testing, both vestibular systems could not be tested separately. Therefore, these test results were described per subject and not per vestibular organ. Vestibular test results not included in the analysis were noted as “missing data,” “unreliable” or “not performed.” “Missing data” was considered if a patient met the criteria to undergo the test, but no measurements were recorded and no reason to not perform the test was described. In case of “unreliable results,” the test was performed, but results were considered unreliable due to several reasons. These reasons included for example for VHIT: artifacts due to excessive blinking, too much resistance against moving of the head, and vision disorders such as strabismus, which influenced the monitoring of the eyes. For rotatory chair testing, unreliable outcomes were mainly caused by movement of the head and/or body or excessive blinking during the test. In the case of cVEMP, the incapability to stabilize the muscle contractions led to unreliable outcomes. A test was considered “not performed” in case it was clearly reported that the test was not performed. After all, in some cases, certain tests were not performed due to the existing protocol at that period of time, or further testing was not possible as the patient was either uncooperative or fatigued.

Demographic data such as age, gender and sensorineural hearing loss classification in accordance with the GENDEAF guidelines (27) were analyzed (Table 1). Additionally, an experienced otorhinolaryngologist (J.W.) classified the patients as having VH (either unilateral or bilateral) or normal vestibular function, based on a combination of the clinical presentation, medical and patient history, and vestibular test results of the patient. Clinical presentation was based on the age when the expected motor milestones, such as crawling or walking, were reached and the perceived clumsiness by the parents of the patient or by the patient him or herself. Concerning the medical history, certain patient-related factors were taken into consideration for assessing pre-test probability of diagnosing vestibular hypofunction. For example, some subjects were diagnosed prior to or during the work-up with a syndrome or disease that was linked to potential vestibular pathology. In addition, certain cases were diagnosed or presented with anomalies of the inner ear determined by computed tomography or magnetic resonance imaging (see appendix table 1). Lastly, the interpretation of the test results took into account whether there was only one (or more) tests classified as abnormal as well as the degree of aberration (i.e., slight abnormality in cVEMP amplitude or latency, combined with a normal motor development could be deemed physiological). This classification was used for the final “expert diagnosis” in this study. In summary, vestibular (hypo-) function was diagnosed after complete assessment of the clinical presentation, medical and patient history, physical examination, vestibular test results and, if performed, diagnostic imaging.

**Table 1:** Patient characteristics.

	Cohort (*N* = 172 ears in 86 patients) *N*
Mean age in years (SD)	5.3 (4.0)
Gender Female Male	40 patients (47%) 46 patients (53%)
Degree of SNHL None (<30 dB) Mild (30–40 dB) Moderate (41–70 dB) Severe (71–95 dB) Profound (>95 dB)	19 ears (11%) 14 ears (8%) 45 ears (26%) 68 ears (40%) 26 ears (15%)
Side of SNHL Bilateral Unilateral	67 patients (78%) 19 patients (22%)

Degree of hearing loss according to GENDEAF guidelines for the better ear.

*N*, absolute number; SNHL, sensorineural hearing loss; dB, decibels.

The combination of test result(s) and expert diagnosis were categorized as true negative (TN), true positive (TP), false negative (FN) or false positive (FP). Subsequently, diagnostic quality was assessed by determining sensitivity, specificity and diagnostic accuracy with the following formulas:


$$\mathrm{Sensitivity}\;\left(\%\right)=\frac{TP}{TP+FN}*100$$

$$\mathrm{Specificity}\;\left(\%\right)=\frac{TN}{TN+FP}*100$$

$$\mathrm{Diagnostic}\;\mathrm{accuracy}\;\left(\%\right)=\frac{TP+TN}{\mathrm{total}}*100$$

TN was recorded as an expert diagnosis of normal vestibular function and normal test result, while TP was recorded as an expert diagnosis of VH and abnormal test result. FN and FP were recorded when the diagnosis by the expert and test did not agree on the outcome, in which case the “expert diagnosis” by the aforementioned otorhinolaryngologist counted as the reference standard. Test outcomes were assessed for individual tests and test combinations of the VHIT, caloric test and cVEMP. Rotatory chair testing (torsion swing) was not considered for determining the diagnostic accuracy in combination with other tests, as this test analyzed both vestibular systems simultaneously and could not be interpreted per vestibular system. In case of determining the diagnostic accuracy of a combination of tests, the following combination of outcome measurements were regarded as either true or false positive and true or false negative: TP indicated that at least one of the performed tests per vestibular system was considered TP as described above; TN if all performed tests were considered TN per vestibular system as described above; FN if at least one of the tests was interpreted as FN and the remaining tests were either FN or TN, and; FP if at least one test was regarded as FP and the remaining tests were either TN or FN.

### 2.4 Statistical analysis

Descriptive statistics and analysis were used to categorize different outcome measurements and demographics of the cohort, and to calculate the diagnostic accuracy (e.g., sensitivity, specificity and diagnostic accuracy). Further statistical analysis of the acquired data was ruled unfeasible due to a number of reasons. Firstly, the aforementioned description of not included test results (‘missing data’, ‘unreliable result’ and ‘not performed’) led to selective loss of data that is considered attrition bias. Overall, the reason for incomplete testing could be accounted to the level of instructability of the individual. A variety of factors influenced this aspect, such as the age, degree of hearing loss and cognitive development. Secondly, diagnosing VH is a partially subjective interpretation of the clinical assessment and vestibular test results as there is no diagnostic tool that confirms this diagnosis objectively. Thirdly, there is an overlap between the outcomes of the individual vestibular tests and the combinations of tests that were formed to determine diagnostic accuracy. Therefore, the assessed tests and combinations of tests are not independent from each other. In addition, the individual tests and test combinations were formed retrospectively and were therefore not standardized and differed in patient characteristics between each other. The partial overlap and inter-group differences resulted in a loss of independence and comparability between the tests and formed combinations, leading to biased and unreliable statistical testing. In summary, comparative group testing takes certain patient, test and group characteristics into consideration and the current methodology did not meet the requirements to perform this type of statistical analysis.

## 3 Results

### 3.1 Patient characteristics

Vestibular testing was performed in 86 patients with uni- or bilateral SNHL (172 ears; 40 girls (47%) and 46 boys (53%)). The mean age was 5.3 years, with a standard deviation of 4.0. Sixty-seven patients (78%) had binaural SNHL and 19 (22%) had unilateral SNHL. Most of the ears (40%) had severe SNHL with a pure tone average of 80 dB (see Table 1).

### 3.2 Vestibular test results

Table 2 presents the results of the vestibular tests in children with SNHL. Vestibular hypofunction was most often demonstrated by the caloric test (56%), and least often by VHIT (20%). The cVEMP and rotatory chair showed the highest percentage of unreliable results in the complete cohort of tested patients. Regarding caloric testing, no unreliable results were found. It was also the test that was least performed compared to the other vestibular tests. Based on these test results combined with the clinical presentation, VH was clinically diagnosed in 44% (38/86) of the patients (Appendix Table 1). Of the 38 patients diagnosed with VH, 24 patients were diagnosed with bilateral VH and 14 with unilateral VH. Of the 24 patients with bilateral VH, 20 had bilateral SNHL and four had unilateral SNHL. Among the 14 patients with unilateral VH, eight had bilateral SNHL and six had unilateral SNHL. Interestingly, eight of the patients with bilateral SNHL showed unilateral VH, and four with unilateral SNHL showed bilateral VH (Appendix Table 2). See Appendix Table 1 for an overview of cases with VH, the side of SNHL and related pathologies. In the group of patients diagnosed with VH, eight showed abnormal test results only for horizontal semicircular canal function tests (VHIT, caloric test or rotatory chair test) while the cVEMP was normal, seven showed abnormal test results only in the saccular function test (cVEMP) while horizontal semicircular canal function tests were normal (appendix table 3). In the remaining 23 patients, abnormal test results were found for both the horizontal semicircular canal and sacculus.

**Table 2:** Abnormal test results (suggestive for vestibular hypofunction) and normal test results (suggestive for normal vestibular function) for each test per vestibular organ tested.

	Abnormal test result *N* (% of total reliable results)	Normal test result *N* (% of total reliable results)	Total reliable results *N* (% of total ears)	Missing data *N* (% of total ears)	Unreliable N (% of total ears)	Not performed *N* (% of total ears)	Total ears *N*
Horizontal semicircular canal function test							
VHIT	16 (20%)	66 (80%)	82 (47%)	34 (20%)	16 (9%)	40 (23%)	172
Caloric test	35 (56%)	29 (44%)	64 (37%)	32 (19%)	0 (0%)	76 (44%)	172
Rotary test*	22 (45%)	27 (55%)	49 (56%)	25 (29%)	12 (14%)	0 (0%)	86
Saccular function test							
cVEMP	28 (32%)	60 (68%)	88 (51%)	30 (17%)	22 (13%)	32 (19%)	172

*Results counted per subject; VHIT, video head impulse test; cVEMP, cervical evoked myogenic potential.

### 3.3 Diagnostic accuracy of vestibular tests in patients with SNHL

Table 3 demonstrates the diagnostic accuracy of the vestibular tests when compared to the expert diagnosis. Sensitivity was highest for the caloric test (94%) compared to the other tests, when performed singularly. The cVEMP (57%) showed the lowest sensitivity of the four included tests. When combining vestibular tests to detect VH, sensitivity increased compared to the sensitivity of each test separately. A combination of VHIT and caloric testing led to a sensitivity of 100%. The remaining combinations (VHIT and cVEMP; cVEMP and caloric test) resulted in sensitivities of 95 and 97%, respectively. Regarding specificity, the highest specificity was found in VHIT (98%) and the lowest in the caloric test (90%). All combinations of tests led to a decrease of the specificity. In the group of patients that successfully completed VHIT, cVEMP and caloric testing, combining all three tests resulted in a sensitivity of 97% and a specificity of 89%. Diagnostic accuracy was the highest for the caloric test with 92% and the lowest for cVEMP with 75%. Combining vestibular tests improved the diagnostic accuracy of all tests.

**Table 3:** Diagnostic quality for each vestibular test per vestibular system for determining vestibular hypofunction.

Vestibular test(s)	TP	FP	TN	FN	Total	Sensitivity	Specificity	Diagnostic accuracy
VHIT	15	1	60	6	82	71%	98%	91%
Caloric test	32	3	27	2	64	94%	90%	92%
Rotary chair*	21	1	17	10	49	68%	94%	78%
cVEMP	26	2	40	20	88	57%	95%	75%
VHIT + caloric test	35	1	8	0	44	100%	89%	98%
VHIT + cVEMP	38	2	22	2	64	95%	92%	94%
Caloric test + cVEMP	32	3	24	1	60	97%	89%	93%
VHIT + caloric test + cVEMP	28	1	8	1	38	97%	89%	95%

*Result counted per subject; TP, true positive, FP, false positive, TN, true negative, FN, false negative, VHIT, video head impulse test; cVEMP, cervical evoked myogenic potential.

The group of patients that successfully completed VHIT, caloric testing and cVEMP, was separately analyzed regarding the diagnostic accuracy (appendix table 4) and compared to the complete cohort. The sensitivity of the caloric test remained similar, while the VHIT and cVEMP scored higher compared to the complete cohort (89 and 88%, respectively). Regarding specificity, an increase was observed for the caloric test (100%) and the VHIT (100%), while the cVEMP showed a small decrease (93%). The diagnostic accuracy noticeably improved for the cVEMP (89%), whereas the caloric test (92%) and VHIT (89%) yielded similar results.

## 4 Discussion

The objective of this study was to evaluate the diagnostic accuracy of several clinically available vestibular tests in children with SNHL, and to provide recommendations for the implementation of vestibular testing of children in clinical practice, to screen for VH. The diagnostic accuracy was highest for the caloric test and VHIT. Combining vestibular tests to detect VH, resulted in a higher diagnostic accuracy and sensitivity, compared to the same characteristics of each test separately. However, specificity decreased when combining tests, but remained >89%. These outcomes were similar for the separate analysis of the patients that underwent all three tests (appendix table 4). Interestingly, adding a third test did not noticeably increase the sensitivity or diagnostic accuracy compared to other combinations of tests. These findings indicate that performing at least two vestibular tests increases the likelihood of a positive and reliable diagnosis of VH in children.

To our knowledge, this is the first study that addresses the diagnostic accuracy of these vestibular tests in children. Nevertheless, sensitivity and specificity rates were described before for some of the tests: VHIT, rotatory chair testing and cVEMP. The caloric test was often considered the gold standard to determine VH, and therefore no sensitivity or specificity rates were previously reported in children (22). Regarding VHIT and cVEMP, the sensitivity and specificity rates were comparable to previous studies (1, 28–36). Regarding rotatory chair testing, sensitivity was comparable to previous literature, in contrast to specificity. This latter was considerably higher in this study (1, 31, 32, 34, 36). It should be mentioned that the sensitivity and specificity was assessed differently in these studies. The aforementioned studies used rotatory chair testing or caloric testing in the same subject as the gold standard. In this study, the ‘expert diagnosis’ was considered the gold standard.

The VHIT demonstrated a high diagnostic accuracy. However, its sensitivity was 71% in the study population. This finding suggests that relying solely on the VHIT may result in underdiagnosis of VH in children who might show VH in other vestibular tests. This discrepancy between results of different vestibular tests is well known in vestibular medicine. It can be related to pathophysiology (e.g., hydrops) or differences in test characteristics (e.g., testing canals or otoliths, susceptibility to artifacts, etc.) (37, 38). The diagnostic accuracy of the cVEMP and rotatory chair test (75 and 78%, respectively) was considerably lower compared to the VHIT (91%) and caloric test (92%). Several explanations could be hypothesized. First, it could be inherent to the tests and their interpretation. Regarding cVEMP, subtle changes in otolith function cannot be detected as a result from the large range in normative data (16, 39). Therefore, VEMPs are often interpreted as ‘absent’ or ‘present’, not taking into account subtle changes in ‘present’ responses (39). This could compromise sensitivity. Regarding rotatory chair testing: the torsion swing test is not able to selectively test one vestibular organ. Unilateral vestibular hypofunction can therefore be missed in compensated vestibular hypofunction (16), decreasing sensitivity. Additionally, responses to the torsion swing test seem to be preserved longer compared to VHIT and caloric testing in patients with bilateral VH (40). Secondly, the caloric test was only performed in an older population (above the age of four). Interpretation of the test outcomes and clinical assessment could therefore be more easily. Thirdly, the number of false negative results noticeably dropped for VHIT and cVEMP, when combined with an additional test. This implies that combining vestibular tests enhances diagnostic accuracy.

**Figure 1:**
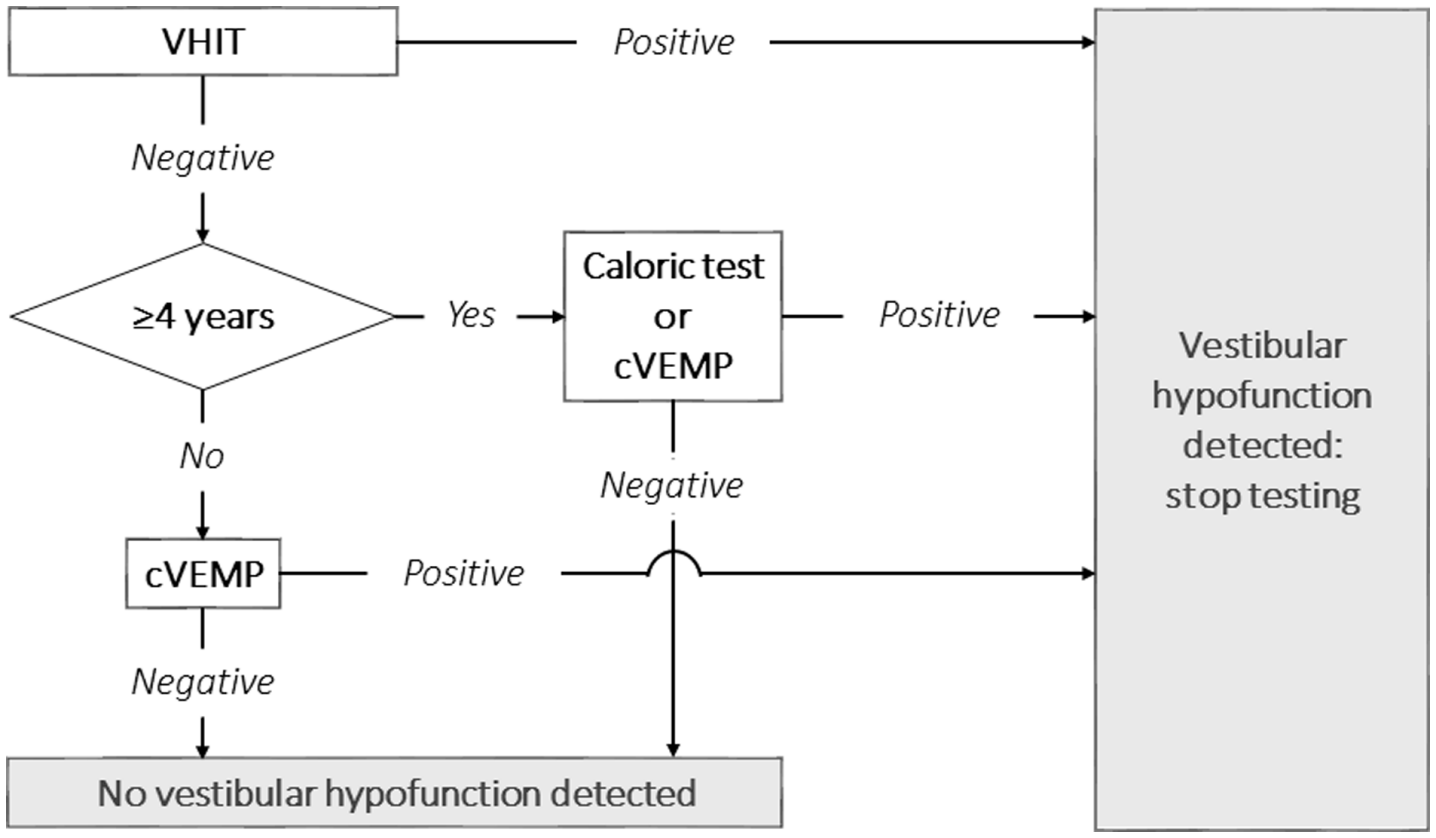
Proposal for a clinical vestibular testing algorithm in children to detect vestibular hypofunction, taking into account the burden of testing and diagnostic accuracy. It should be noted that the velocity step test was not included in this study, and therefore not included in this proposal. VHIT, video head impulse test; cVEMP, cervical vestibular evoked myogenic potential.

As such, this study emphasizes the importance of access to a multimodal vestibular testing battery. After all, combining vestibular tests increased diagnostic accuracy. Taking into account the findings of this study, a diagnostic algorithm could be proposed, as illustrated in Figure 1. The concept of this diagnostic algorithm is based on the burden of testing and diagnostic accuracy of the included tests. It could therefore be considered to start with VHIT, since the burden of VHIT is low, the test is relatively quick, and it has a high specificity (29). In case VH is detected, no additional vestibular tests might be needed. In case no VH is detected, cVEMP could be performed subsequently on children below the age of four. In case no VH is detected and children are >4 years old, the caloric test or cVEMP could be performed. Combining the caloric test with VHIT yields a higher diagnostic accuracy than combing cVEMP with VHIT. However, the caloric test has the highest burden of testing and is more time consuming than cVEMP (20, 38, 41). As noted in Table 3 and appendix table 4, adding a third test did not substantially improve diagnostic accuracy. Therefore, a third vestibular test could be considered redundant if the primary goal of testing is to detect VH as efficiently as possible. The torsion swing test was not included in this algorithm, since no children demonstrated only a true positive torsion swing test and false negative results in the other tests. The value of the torsion swing test to detect VH was therefore considered minimal, compared to the other tests. However, it should be noted that another rotatory chair test, the velocity step test, was not included in this study. This test is able to more selectively test one vestibular organ, than the torsion swing test (16). Future research should determine the value of the velocity step test in this diagnostic algorithm.

The prevalence of VH in this study was comparable to earlier reports (4, 6, 13, 22, 42, 43). The lack of a gold standard creates a wide range in prevalence of VH, since some of the studies based the diagnosis of VH on a single vestibular test. This study shows the limitations of using a single test modality as this leads to a higher chance of misdiagnosing children with normal vestibular function. Moreover, the relevance of performing a single test that addresses the otoliths, such as the cVEMP, is debatable, as the clinical presentation of isolated otolith dysfunction is not yet clearly understood. Furthermore, preserved otolith responses do not exempt other vestibular structures of being affected (44–46). Regarding hearing status and VH, no correlation was found between the side (s) of SNHL, the degree of SNHL and the side(s) of VH. This shows the importance to test vestibular function bilaterally in children regardless of unilateral or bilateral SNHL (47).

As mentioned above, this study has a number of limitations. Firstly, due to the attention span of children it was difficult to execute all the tests (correctly) in all children. Consequently, a certain number of the tests were scored as ‘not performed’ or ‘missing data’. This hampers the comparison of the diagnostic value for each individual test and leads to attrition bias. Finally, no gold standard is yet available to test VH in children. Hence, the diagnosis was based on a combination of the test results and the clinical examination of the patient interpreted by an expert in pediatric vestibular disorders. The comparison between the diagnosis and the initial test result to calculate the diagnostic accuracy of each vestibular test can be regarded as circular reasoning, as the diagnosis is partially based on the test results itself. To decrease this variable factor for diagnosing vestibular function, normative values were acquired from healthy children. These values helped to establish a diagnosis based on normative data and made the diagnosis less dependent on subjective interpretation. These limitations underscore the disadvantages of retrospective analysis, while also highlighting the current challenges in testing and assessing children’s balance.

## 5 Conclusion

Vestibular testing is feasible and VH is highly prevalent in children with SNHL. The acquired data suggests a testing approach that balances the duration of testing, burdening of the child and diagnostic accuracy. A proposed diagnostic algorithm, based on these factors, recommends starting with VHIT, followed by cVEMP for children under the age of four, and caloric testing for older children if VH is not confirmed with the first test. Performing a third test is redundant as the diagnostic accuracy does not improve substantially. Early recognition of VH holds promise for prompt interventions, such as vestibular rehabilitation therapy to support motor development or adequate decision making in surgical interventions, such as cochlear or vestibular implantation (15). However, vestibular testing in children remains challenging due to population-related factors and testing limitations. Hopefully, this will improve through standardization of testing and increasing knowledge of VH in children with SNHL.

## Data availability statement

The raw data supporting the conclusions of this article will be made available by the authors, without undue reservation.

## Ethics statement

The studies involving humans were approved by the Ethics Committee of the Maastricht University Medical Center and Ethics Committee of the Antwerp University Hospital. The studies were conducted in accordance with the local legislation and institutional requirements. Written informed consent for participation in this study was provided by the participants’ legal guardians/next of kin.

## Author contributions

MG: Writing – original draft, Writing – review & editing. TH: Writing – original draft. AB: Writing – review & editing. VR: Writing – review & editing. RB: Writing – original draft, Writing – review & editing. JW: Writing – original draft, Writing – review & editing.

## References

[1] Cushing S Papsin BC Rutka JA James AL Gordon KA. Evidence of vestibular and balance dysfunction in children with profound sensorineural hearing loss using cochlear implants. Laryngoscope. (2008) 118:1814–23. doi: 10.1097/MLG.0b013e31817fadfa, PMID: 18758383

[2] Nandi R Luxon LM. Development and assessment of the vestibular system. Int J Audiol. (2008) 47:566–77. doi: 10.1080/14992020802324540 18821226

[3] Rine RM. Growing evidence for balance and vestibular problems in children. Audiol Med. (2009) 7:138–42. doi: 10.1080/16513860903181447

[4] de Kegel A Maes L Baetens T Dhooge I van Waelvelde H. The influence of a vestibular dysfunction on the motor development of hearing-impaired children. Laryngoscope. (2012) 122:2837–43. doi: 10.1002/lary.23529, PMID: 22990988

[5] Cushing SL Papsin BC Rutka JA James AL Blaser SL Gordon KA. Vestibular end-organ and balance deficits after meningitis and cochlear implantation in children correlate poorly with functional outcome. Otol Neurotol. (2009) 30:488–95. doi: 10.1097/MAO.0b013e31819bd7c8 19395989

[6] Maes L de Kegel A van Waelvelde H Dhooge I. Rotatory and collic vestibular evoked myogenic potential testing in normal-hearing and hearing-impaired children. Ear Hear. (2014) 35:e21–32. doi: 10.1097/AUD.0b013e3182a6ca91, PMID: 24556969

[7] Janky KL Thomas MLA High RR Schmid KK Ogun OA. Predictive factors for vestibular loss in children with hearing loss. Am J Audiol. (2018) 27:137–46. doi: 10.1044/2017_AJA-17-0058, PMID: 29482202 PMC6105082

[8] Angeli S. Value of vestibular testing in young children with sensorineural hearing loss. Arch Otolaryngol Head Neck Surg. (2003) 129:478–82. doi: 10.1001/archotol.129.4.478 12707199

[9] Lacroix E Edwards MG de Volder A Noël MP Rombaux P Deggouj N. Neuropsychological profiles of children with vestibular loss. J Vestib Res. (2020) 30:25–33. doi: 10.3233/VES-200689, PMID: 32083606

[10] Bigelow RT Agrawal Y. Vestibular involvement in cognition: visuospatial ability, attention, executive function, and memory. J Vestib Res. (2015) 25:73–89. doi: 10.3233/VES-150544, PMID: 26410672

[11] Braswell J Rine RM. Evidence that vestibular hypofunction affects reading acuity in children. Int J Pediatr Otorhinolaryngol. (2006) 70:1957–65. doi: 10.1016/j.ijporl.2006.07.013, PMID: 16945429

[12] Rine RM Braswell J Fisher D Joyce K Kalar K Shaffer M. Improvement of motor development and postural control following intervention in children with sensorineural hearing loss and vestibular impairment. Int J Pediatr Otorhinolaryngol. (2004) 68:1141–8. doi: 10.1016/j.ijporl.2004.04.007, PMID: 15302144

[13] Kotait MA Moaty AS Gabr TA. Vestibular testing in children with severe-to-profound hearing loss. Int J Pediatr Otorhinolaryngol. (2019) 125:201–5. doi: 10.1016/j.ijporl.2019.07.015, PMID: 31401454

[14] Chen X Chen X Zhang F Qin Z. Influence of cochlear implantation on vestibular function. Acta Otolaryngol. (2016) 136:655–9. doi: 10.3109/00016489.2016.1154186 27008103

[15] van de Berg R Ramos A van Rompaey V Bisdorff A Perez-Fornos A Rubinstein JT. The vestibular implant: opinion statement on implantation criteria for research. J Vestib Res. (2020) 30:213–23. doi: 10.3233/VES-200701, PMID: 32651339 PMC9249290

[16] Starkov D Strupp M Pleshkov M Kingma H van de Berg R. Diagnosing vestibular hypofunction: an update. J Neurol. (2021) 268:377–85. doi: 10.1007/s00415-020-10139-4, PMID: 32767115 PMC7815536

[17] Fife TD Tusa RJ Furman JM Zee DS Frohman E Baloh RW. Assessment: vestibular testing techniques in adults and children: report of the therapeutics and technology assessment Subcommittee of the American Academy of neurology. Neurology. (2000) 55:1431–41. doi: 10.1212/WNL.55.10.1431 11094095

[18] Aw S Haslwanter T Fetter M Heimberger J Todd MJ. Contribution of the vertical semicircular canals to the caloric nystagmus. Acta Otolaryngol. (1998) 118:618–27. doi: 10.1080/00016489850183089, PMID: 9840495

[19] van Esch BF Nobel-Hoff GEAJ van Benthem PPG van der Zaag-Loonen HJ Bruintjes TD. Determining vestibular hypofunction: start with the video-head impulse test. Eur Arch Otorrinolaringol. (2016) 273:3733–9. doi: 10.1007/s00405-016-4055-9, PMID: 27113255

[20] Schmid-Priscoveanu A BÃ¶hmer A Obzina H Straumann D. Caloric and search-coil head-impulse testing in patients after vestibular neuritis. J Assoc Res Otolaryngol. (2001) 2:72–8. doi: 10.1007/s101620010060, PMID: 11545152 PMC3201096

[21] Janky KL Rodriguez AI. Quantitative vestibular function testing in the pediatric population. Semin Hear. (2018) 39:257–74. doi: 10.1055/s-0038-1666817, PMID: 30038454 PMC6054588

[22] Verbecque E Marijnissen T de Belder N van Rompaey V Boudewyns A van de Heyning P. Vestibular (dys)function in children with sensorineural hearing loss: a systematic review. Int J Audiol. (2017) 56:361–81. doi: 10.1080/14992027.2017.1281444, PMID: 28264605

[23] Wiener-Vacher SR Wiener SI. Video head impulse tests with a remote camera system: normative values of Semicircular Canal Vestibulo-ocular reflex gain in infants and children. Front Neurol. (2017) 8:434. doi: 10.3389/fneur.2017.00434, PMID: 28936193 PMC5594068

[24] Verrecchia L Galle Barrett K Karltorp E. The feasibility, validity and reliability of a child friendly vestibular assessment in infants and children candidates to cochlear implant. Int J Pediatr Otorhinolaryngol. (2020) 135:110093. doi: 10.1016/j.ijporl.2020.110093, PMID: 32422368

[25] Jongkees LB Maas JP Philipszoon AJ. Clinical nystagmography. A detailed study of electro-nystagmography in 341 patients with vertigo. Pract Otorhinolaryngol. (1962) 24:65–93. PMID: 14452374

[26] Martens S Dhooge I Dhondt C Vanaudenaerde S Sucaet M Rombaut L. Vestibular infant screening (VIS)-Flanders: results after 1.5 years of vestibular screening in hearing-impaired children. Sci Rep. (2020) 10:21011. doi: 10.1038/s41598-020-78049-z, PMID: 33273502 PMC7713061

[27] M M van CAMP G NEWTON V N G F D PARVING A. Recommendations for the description of genetic and audiological data for families with nonsyndromic hereditary hearing impairment. Audiol Med. (2003) 1:148–50. doi: 10.1080/16513860301713

[28] Hamilton SS Zhou G Brodsky JR. Video head impulse testing (VHIT) in the pediatric population. Int J Pediatr Otorhinolaryngol. (2015) 79:1283–7. doi: 10.1016/j.ijporl.2015.05.033, PMID: 26066850

[29] Kim KS Jung YK Hyun KJ Kim MJ Kim HJ. Usefulness and practical insights of the pediatric video head impulse test. Int J Pediatr Otorhinolaryngol. (2020) 139:110424. doi: 10.1016/j.ijporl.2020.110424, PMID: 33039719

[30] Janky KL Givens D. Vestibular, visual acuity, and balance outcomes in children with Cochlear implants: a preliminary report. Ear Hear. (2015) 36:e364–72. doi: 10.1097/AUD.0000000000000194, PMID: 26182202 PMC5113611

[31] Inoue A Iwasaki S Ushio M Chihara Y Fujimoto C Egami N. Effect of vestibular dysfunction on the development of gross motor function in children with profound hearing loss. Audiol Neurootol. (2013) 18:143–51. doi: 10.1159/000346344, PMID: 23392310

[32] Kaga K Shinjo Y Jin Y Takegoshi H. Vestibular failure in children with congenital deafness. Int J Audiol. (2008) 47:590–9. doi: 10.1080/14992020802331222 18821229

[33] Jacot E van den Abbeele T Debre HR Wiener-Vacher SR. Vestibular impairments pre- and post-cochlear implant in children. Int J Pediatr Otorhinolaryngol. (2009) 73:209–17. doi: 10.1016/j.ijporl.2008.10.024, PMID: 19101044

[34] Shinjo Y Jin Y Kaga K. Assessment of vestibular function of infants and children with congenital and acquired deafness using the ice-water caloric test, rotational chair test and vestibular-evoked myogenic potential recording. Acta Otolaryngol. (2007) 127:736–47. doi: 10.1080/00016480601002039, PMID: 17573570

[35] Tribukait A Brantberg K Bergenius J. Function of semicircular canals, utricles and saccules in deaf children. Acta Otolaryngol. (2004) 124:41–8. doi: 10.1080/00016480310002113, PMID: 14977077

[36] Zagolski O. Vestibular system in infants with hereditary nonsyndromic deafness. Otol Neurotol. (2007) 28:1053–5. doi: 10.1097/MAO.0b013e31815145e9 17898670

[37] McGarvie LA Curthoys IS MacDougall HG Halmagyi GM. What does the head impulse test versus caloric dissociation reveal about vestibular dysfunction in Meniere's disease? Ann N Y Acad Sci. (2015) 1343:58–62. doi: 10.1111/nyas.12687, PMID: 25721760

[38] van de Berg R van Tilburg M Kingma H. Bilateral vestibular hypofunction: challenges in establishing the diagnosis in adults. ORL J Otorhinolaryngol Relat Spec. (2015) 77:197–218. doi: 10.1159/000433549, PMID: 26366566

[39] Rosengren SM Welgampola MS Taylor RL. Vestibular-evoked myogenic potentials in bilateral Vestibulopathy. Front Neurol. (2018) 9:252. doi: 10.3389/fneur.2018.00252, PMID: 29719527 PMC5913369

[40] Lucieer F Vonk P Guinand N Stokroos R Kingma H van de Berg R. Bilateral vestibular hypofunction: insights in etiologies, Clinical subtypes, and diagnostics. Front Neurol. (2016) 7:26. doi: 10.3389/fneur.2016.00026 26973594 PMC4777732

[41] Dhondt C Dhooge I Maes L. Vestibular assessment in the pediatric population. Laryngoscope. (2019) 129:490–3. doi: 10.1002/lary.27255 30394531

[42] Gadsboll E Erbs AW Hougaard DD. Prevalence of abnormal vestibular responses in children with sensorineural hearing loss. Eur Arch Otorrinolaringol. (2022) 279:4695–707. doi: 10.1007/s00405-021-07241-2, PMID: 35156132

[43] Martens S Dhooge I Dhondt C Vanaudenaerde S Sucaet M van Hoecke H. Three years of vestibular infant screening in infants with sensorineural hearing loss. Pediatrics. (2022) 150:e2021055340. doi: 10.1542/peds.2021-055340, PMID: 35698886

[44] de Chua KW Yuen HW Low DAYM Kamath S. Proposal on the diagnostic criteria of definite isolated otolith dysfunction. J Audiol Otol. (2021) 25:59–60. doi: 10.7874/jao.2020.00535, PMID: 33327705 PMC7835430

[45] Park HG Lee JH Oh SH Park MK Suh MW. Proposal on the diagnostic criteria of definite isolated otolith dysfunction. J Audiol Otol. (2019) 23:103–11. doi: 10.7874/jao.2018.00374, PMID: 30562878 PMC6468282

[46] Suh MW Murofushi T. Response: proposed diagnostic criteria for definite isolated otolith dysfunction. J Audiol Otol. (2021) 25:61–3. doi: 10.7874/jao.2020.00661, PMID: 33327704 PMC7835435

[47] Sokolov M Gordon KA Polonenko M Blaser SI Papsin BC Cushing SL. Vestibular and balance function is often impaired in children with profound unilateral sensorineural hearing loss. Hear Res. (2019) 372:52–61. doi: 10.1016/j.heares.2018.03.032, PMID: 29655975

