# Supplementary material for: Vestibular assessment in children with sensorineural hearing loss: diagnostic accuracy and proposal for a diagnostic algorithm
Source: Front Neurol. 2024 Feb 1;15:1349554. doi: 10.3389/fneur.2024.1349554 (PMC10867167; doi:10.3389/fneur.2024.1349554)
Supplement: Supplementary file 1 [file Table_1.DOCX]

Table 1: Cases of vestibular hypofunction in the cohort and the relation between the side sensorineural hearing loss and side of vestibular hypofunction.

| Case | SNHL | dB AD | dB AS | VH | Pathology |
| --- | --- | --- | --- | --- | --- |
| 1 | ADS | 109 | 91 | ADS | Congenital cytomegalovirus |
| 2 | ADS | 93 | 90 | ADS | Idiopathic |
| 3 | ADS | 89 | 85 | ADS | Idiopathic |
| 4 | ADS | 97 | 93 | ADS | Beckwith-Wiedemann syndrome |
| 5 | ADS | 107 | 97 | ADS | Congenital cytomegalovirus |
| 6 | ADS | 52 | 57 | ADS | Idiopathic |
| 7 | ADS | 116 | 116 | ADS | Waardenburg syndrome type 2D (mutation SOX10-gene) |
| 8 | ADS | 57 | 85 | ADS | Congenital cytomegalovirus |
| 9 | ADS | 118 | 118 | ADS | Homozygote mutation Myo15A-gene |
| 10 | ADS | >120 | >120 | ADS | Waardenburg syndrome type 1 (mutation PAX3-gene) |
| 11 | ADS | 35 | >120 | ADS | Congenital CMV |
| 12 | ADS | 62 | 67 | ADS | Mutation SIX1-gene |
| 13 | ADS | >120 | >120 | ADS | Usher type 1 |
| 14 | ADS | >120 | >120 | ADS | Idiopathic |
| 15 | ADS | >120 | >120 | ADS | Idiopathic |
| 16 | ADS | 107 | 118 | ADS | Microtia right side with suspected Guion-Almeida syndrome |
| 17 | ADS | >120 | >120 | ADS | Waardenburg syndrome type 2E |
| 18 | ADS | 69 | >120 | ADS | CHARGE syndrome and congenital cytomegalovirus |
| 19 | ADS | 78 | 89 | ADS | Idiopathic |
| 20 | ADS | 118 | 118 | ADS | Homozygote mutation Myo15A-gene |
| 21 | ADS | 90 | 95 | AD | Mutation BCAP31-gene |
| 22 | ADS | 97 | 101 | AD | Auditory Neuropathy Spectrum Disorder |
| 23 | ADS | 61 | 60 | AS | Mutation OTOGL-gene |
| 25 | ADS | 82 | 84 | AD | Idiopathic |
| 25 | ADS | 35 | 35 | AS | Aplasia left horizontal semicircular canal |
| 26 | ADS | >120 | 46 | AS | Congenital cytomegalovirus |
| 27 | ADS | 65 | 84 | AS | Homozygote mutation GJB2-gene |
| 28 | ADS | 32 | 115 | AD | Congenital cytomegalovirus |
| 29 | AD | 46 |  | ADS | Idiopathic |
| 30 | AS |  | 70 | ADS | CHARGE syndrome |
| 31 | AS |  | 55 | ADS | Idiopathic |
| 32 | AD | >120 |  | ADS | Congenital cytomegalovirus |
| 33 | AD | 51 |  | AD | Mutation KCNQ2-gene |
| 34 | AS | >120 | >120 | AS | Pneumococcal meningitis |
| 35 | AD | >120 |  | AD | Aplasia right cochlear nerve |
| 36 | AD | 30 |  | AD | Idiopathic |
| 37 | AD | 80 |  | AD | Vestibular malformation right with enlarged vestibular aqueduct |
| 38 | AD | 43 |  | AD | Idiopathic |

**SNHL= sensorineural hearing loss; dB= decibel; AD= right ear; AS= left ear; VH= vestibular hypofunction; ADS= both ears.**

Table 2: relation between unilateral or bilateral sensorineural hearing loss and vestibular hypofunction in children

| SNHL (>30 dB) | Unilateral VH | Bilateral VH |
| --- | --- | --- |
| Unilateral | 6 | 4 |
| Bilateral | 8 | 20 |

**SNHL= sensorineural hearing loss; dB= decibel; VH= vestibular hypofunction**

Table 3: Comparison of test results per vestibular component in children with vestibular hypofunction.

|  | Normal HSCC function | HSCC hypofunction |
| --- | --- | --- |
| Normal saccular function | x | 8 |
| Saccular hypofunction | 7 | 23 |

**HSCC= horizontal semicircular canal; Saccular (hypo-)function= cVEMP; HSCC (hypo-)function= video head impulse test and/or caloric test and/or rotatory chair.**

Table 4: diagnostic quality of single tests in subgroup with all three tests performed per vestibular system.

| \| Vestibular tests \| TP \| FP \| TN \| FN \| Total \| Sensitivity \| Specificity \| Diagnostic accuracy \| \| --- \| --- \| --- \| --- \| --- \| --- \| --- \| --- \| --- \| \| VHIT \| 26 \| 0 \| 10 \| 3 \| 38 \| 89% \| 100% \| 94% \| \| Caloric test \| 27 \| 0 \| 9 \| 2 \| 38 \| 93% \| 100% \| 95% \| \| cVEMP \| 21 \| 1 \| 13 \| 3 \| 38 \| 88% \| 93% \| 89% \| \| VHIT + caloric test \| 27 \| 0 \| 10 \| 1 \| 38 \| 96% \| 100% \| 97% \| \| VHIT + cVEMP \| 26 \| 0 \| 10 \| 1 \| 38 \| 96% \| 100% \| 97% \| \| Caloric test + cVEMP \| 27 \| 0 \| 10 \| 1 \| 38 \| 96% \| 100% \| 97% \| \| VHIT + caloric test + cVEMP \| 28 \| 1 \| 8 \| 1 \| 38 \| 97% \| 89% \| 95% \|   TP= true positive; FP= false positive; TN= true negative; FN= false negative; VHIT= video head impulse test; cVEMP= cervical evoked myogenic potential. |
| --- | --- | --- | --- | --- | --- | --- | --- | --- | --- | --- | --- | --- | --- | --- | --- | --- | --- | --- | --- | --- | --- | --- | --- | --- | --- | --- | --- | --- | --- | --- | --- | --- | --- | --- | --- | --- | --- | --- | --- | --- | --- | --- | --- | --- | --- | --- | --- | --- | --- | --- | --- | --- | --- | --- | --- | --- | --- | --- | --- | --- | --- | --- | --- | --- | --- | --- | --- | --- | --- | --- | --- | --- |
